# ICD Shock, Not Ventricular Fibrillation, Causes Elevation of High Sensitive Troponin T after Defibrillation Threshold Testing—The Prospective, Randomized, Multicentre TropShock-Trial

**DOI:** 10.1371/journal.pone.0131570

**Published:** 2015-07-24

**Authors:** Verena Semmler, Jürgen Biermann, Bernhard Haller, Clemens Jilek, Nikolaus Sarafoff, Carsten Lennerz, Hrvoje Vrazic, Bernhard Zrenner, Stefan Asbach, Christof Kolb

**Affiliations:** 1 Deutsches Herzzentrum München, Klinik für Herz- und Kreislauferkrankungen, Abteilung für Elektrophysiologie, Faculty of Medicine, Technische Universität München, Munich, Germany; 2 Cardiology and Angiology I, Heart Center, Freiburg University, Freiburg, Germany; 3 Klinikum rechts der Isar, Institut für Medizinische Statistik und Epidemiologie, Technische Universität, Munich, Germany; 4 Schön Klinik Starnberger See, Kardiologie, Starnberg, Germany; 5 Medizinische Klinik I und Poliklinik, Klinikum der Ludwig-Maximilians-Universität, Munich, Germany; 6 University Hospital Dubrava, Division of Cardiology, Department of Internal Medicine, Zagreb, Croatia; 7 Krankenhaus Landshut-Achdorf, Medizinische Klinik I, Kardiologie, Landshut, Germany; San Raffaele Scientific Institute, ITALY

## Abstract

**Background:**

The placement of an implantable cardioverter defibrillator (ICD) has become routine practice to protect high risk patients from sudden cardiac death. However, implantation-related myocardial micro-damage and its relation to different implantation strategies are poorly characterized.

**Methods:**

A total of 194 ICD recipients (64±12 years, 83% male, 95% primary prevention of sudden cardiac death, 35% cardiac resynchronization therapy) were randomly assigned to one of three implantation strategies: (1) ICD implantation without any defibrillation threshold (DFT) testing, (2) estimation of the DFT without arrhythmia induction (modified “upper limit of vulnerability (ULV) testing”) or (3) traditional safety margin testing including ventricular arrhythmia induction. High-sensitive Troponin T (hsTnT) levels were determined prior to the implantation and 6 hours after.

**Results:**

All three groups showed a postoperative increase of hsTnT. The mean delta was 0.031±0.032 ng/ml for patients without DFT testing, 0.080±0.067 ng/ml for the modified ULV-testing and 0.064±0.056 ng/ml for patients with traditional safety margin testing. Delta hsTnT was significantly larger in both of the groups with intraoperative ICD testing compared to the non-testing strategy (p≤0.001 each). There was no statistical difference in delta hsTnT between the two groups with intraoperative ICD testing (p = 0.179).

**Conclusion:**

High-sensitive Troponin T release during ICD implantation is significantly higher in patients with intraoperative ICD testing using shock applications compared to those without testing. Shock applications, with or without arrhythmia induction, did not result in a significantly different delta hsTnT. Hence, the ICD shock itself and not ventricular fibrillation seems to cause myocardial micro-damage.

**Trial Registration:**

ClinicalTrials.gov NCT01230086

## Introduction

In high-risk patients the implantation of an implantable cardioverter defibrillator (ICD) is the treatment of choice for primary and secondary prevention of sudden cardiac death. To confirm the proper function of the ICD system, intraoperative defibrillation threshold (DFT) testing is widely used [1–2]. Typically, ventricular fibrillation is induced twice by the application of shocks on the T-wave. Termination of the induced arrhythmia with 10 Joule (J) below the maximum output energy of the device is considered to be an adequate safety margin.

However, in recent years the need for a traditional safety margin testing has been questioned [2–4]. One reason for this is the suspicion that ventricular fibrillation and ICD shocks, inherent to the safety margin testing, may cause myocardial damage [5–7]. Several non-randomized studies have shown an elevation of cardiac enzymes measured after ICD implantation with intraoperative DFT testing or after pre-hospital-discharge testing [7–9]. However, the reason for the elevation of cardiac enzymes after DFT testing and also after spontaneous appropriate ICD shocks remains unclear [10–11]. A potential cause of myocardial micro-damage might be that ventricular fibrillation causes myocardial ischemia and consequently an increase in cardiac enzyme levels, or the damage might be directly related to the shock itself. Neither the effect of induced ventricular fibrillation nor the effect of an ICD shock itself on human myocardial tissue have been studied entirely [12]. Therefore, the TropShock trial intends to characterize changes in levels of cardiac enzymes after different intraoperative ICD testing modes. In particular, it aims to elucidate whether induced ventricular fibrillation or the ICD shock itself causes myocardial damage.

## Methods

The study protocol was approved by the ethics committee of the Technical University of Munich as leading ethics committee for the Deutsches Herzzentrum München and Klinikum Landshut (approval number 2869/10; date 07/30/2010), as well as approved by the ethics committee of Freiburg: Albert-Ludwigs-Universität Freiburg, approval number 271/10; date 08/31/2010) and complied with conditions laid out by the declaration of Helsinki. All patients gave their written informed consent prior to study inclusion.

### Study population

The TropShock trial was a prospective, randomized, multi-center trial, which aimed at patients receiving the de-novo implantation of ICD therapy for the primary or secondary prevention of sudden cardiac death; including patients with cardiac resynchronization therapy (CRT) (ClinicalTrials.gov identifier: NCT01230086; for logistic reasons the trial was registered after the recruitment began; the authors confirm that all ongoing and related trials for this intervention are registered). Inclusion criteria were the left-sided placement of an ICD capable of delivering at least 35 J for cardioversions or defibrillations and the intention to place an active fixation defibrillation lead in the apical region of the right ventricle. Patients with any of the following criteria were excluded:

- myocardial infarction, percutaneous coronary intervention, resuscitation or cardiac surgery four weeks prior to ICD implantation,
- coronary artery disease with an indication of revascularization,
- presence of intra-cardiac thrombi,
- contraindication for the induction of ventricular fibrillation or the application of shocks,
- atypical placement of the ventricular lead requiring defibrillation testing,
- right-sided placement of the ICD,
- planned external cardioversion of atrial tachyarrhythmias,
- lead revision or lead extraction,
- upgrade of an pre-existing ICD to a CRT-ICD,
- ASA (American Society of Anesthesiologists) status ≥4,
- inability to give written informed consent, age <18 years.

### Study protocol

Between June 2010 and August 2012 patients were randomly assigned to one of the following three implantation strategies: (1) implantation only (no arrhythmia induction or shock application) (2) implantation with DFT estimation by a modified upper limit of vulnerability testing (implantation plus shock application) or (3) traditional implantation strategy including the induction of ventricular tachyarrhythmias with safety margin testing for the DFT. Random patient allocation was performed by sealed envelopes on a 2:2:1 basis stratified by centre and by CRT versus non-CRT ICD systems.

### Study endpoints

The primary study endpoint was the level of myocardial micro-damage assessed by the delta in the serum high sensitive Troponin T levels (hsTnT) calculated from the difference between the level 6 hours after ICD implantation and the preoperative baseline value.

Pre-specified secondary endpoints included the delta in the serum creatinkinase (total and MB fraction) during the same observational period, and the correlation between the delta hsTnT levels and the procedure time, the number of intra-operative lead repositionings, the underlying cardiac disease and the left ventricular ejection fraction.

All endpoints were evaluated according to the intention to treat principle.

### Implantation procedure and testing methods

Blood samples were drawn before the implantation procedure to determine the baseline serum levels of hsTnT (Elecsys high sensitive Troponin T, Roche diagnostics, Rotkreuz, Switzerland), creatinkinase (total and MB fraction), creatinine and urea. All implantation procedures were performed under analgosedation with the applied drugs left to the discretion of the respective physician. Vital parameters were continuously monitored with the aim of maintaining sedation levels of 3 to 4 according to the Ramsay scale [13] during the whole implantation procedure. Transvenous ICD implantation followed institutional standards with device placement either in a left-sided subpectoral or subcutaneous pocket and with the right ventricular lead positioned in the right ventricular apex. Right atrial and left ventricular leads–where applicable–were also implanted according to institutional standards. After achieving adequate values for the sensing and pacing threshold of the leads, the procedure was continued according to the individual’s randomization assignment.

In patients randomized to “*no defibrillation threshold estimation”*, the pocket was closed and the patient left the operation room without further DFT testing.

In patients randomized to “*implantation with shock application*”, a modified upper limit of vulnerability testing was performed. For this, three ICD shocks at three different energy levels were administered to the ascending part of the T wave. The concept of upper limit of vulnerability testing is based on the observed correlation between the individual DFT and an individual energy amount applied to the vulnerable phase of the repolarization, above which no arrhythmia can be induced [14]. Modified protocols for the upper limit of vulnerability limit the number of arrhythmia inductions [15–17] and we further modified the protocol to allow an estimation of the DFT without inducing ventricular tachyarrhythmias. For this, the energies applied to the heart during the ascending part of the T-wave were carefully selected to achieve a DFT which would equal that achieved by 10 J safety margin testing. The energy was calculated to add up to the same as that which would be delivered cumulatively during traditional 10 J safety margin testing. Finally, energies were adjusted to the different energy levels applicable to the devices of different manufacturers (Table 1).

**Table 1:** Cumulative shock energy.

Manufacturer		Medtronic	St. Jude Medical	Biotronik	Boston Scientific	Sorin
	Maximal shock energy of the device (Joule [J])	35	36	40	41	42
	Shock 1 [J]	1+25	1+25	1+30	1+31	1+32
**Safety margin testing**	Shock 2 [J]	1+25	1+25	1+30	1+31	1+32
	**Σ energy [J]**	**52**	**52**	**62**	**64**	**66**
	Shock 1 [J]	22	22,5	26	27	28
**Upper limit of vulnerability testing**	Shock 2 [J]	18	17,5	22	23	22
	Shock 3 [J]	12	12,5	14	14	16
	**Σ energy [J]**	**52**	**52,5**	**62**	**64**	**66**

Cumulative shock energy applied on the myocardium during safety margin testing or upper limit of vulnerability testing according to randomization and adjusted to the different energy levels applicable to the devices of different manufacturers.

For pure study reasons, a shock coinciding with the R-wave would have been sufficient but potentially unethical. Therefore, the more sophisticated method of the modified upper limit of vulnerability testing was chosen to provide the patient with some estimation of his/her DFT in the setting of our clinical study.

In patients randomized to “*the implantation of the device followed by traditional 10 J safety margin testing”* ventricular fibrillation was twice induced by a shock on T (1 J) and was terminated by a shock 10 J below the maximal available energy of the respective ICD (Table 1).

For the evaluation of hsTnT (primary endpoint), CK and CK-MB levels, further blood samples were taken 6 hours after ICD testing. In patients randomized to “*no testing at all”* the samples were taken at the same time intervals and the “induction time” was presumed to be one minute before the start of the intra-cutaneous suture.

### Procedure in case of deviations from the test protocol

All investigators were repeatedly educated to adhere strictly to the test protocol and to the randomized group. However, certain specific circumstances that could obviate the strict adherence to the testing protocol had been anticipated and recommendations for their management had been given in advance.

In cases in which the apical target region for the right ventricular lead could not be reached or yielded unsatisfactory results for the lead parameters, the implanter could select other pacing sites and it was recommended that traditional safety margin testing should be performed. In the case of ventricular arrhythmia induction during modified upper limit of vulnerability testing, arrhythmia termination had to be attempted at 10 J below the maximal energy provided by the respective device. The further procedure was then left to the discretion of the implanting physician. In the case of atrial arrhythmia induction during modified upper limit of vulnerability testing or during traditional safety margin testing, the implanters were advised to wait 5–10 minutes for spontaneous conversion to sinus rhythm and as a second step to attempt a pharmacological cardioversion (both in order to avoid further shocks as far as possible). If atrial tachyarrhythmias persisted, the implanters were free to decide on an electrical cardioversion at the end of the procedure. If ventricular fibrillation was not inducible (safety margin testing group only) the implanters were only allowed to stop the testing procedure after the application of different coupling intervals for the T-wave shock or the application of a ventricular burst pacing at 50 Hz.

All deviations from the original ICD test protocol were recorded. The analyses of the pre-specified endpoints were carried out applying the intention-to-treat principle but an additional analysis based on per protocol treatment was run.

### Statistics

Because there were no published data available on hsTnT release in relation to ICD implantations, historic data from patients supplied with ICDs in the German Heart Centre were used for sample size calculation. Data from the most recent 30 patients (the most recent 10 patients without intraoperative ICD testing, with modified upper limit of vulnerability testing and traditional safety margin testing, respectively) for which there were available values of hsTnT levels before and after ICD implantation were analysed. The patients did not differ relevantly in their baseline characteristics and the following mean delta ± standard deviations in hsTnT levels were observed: implantation without ICD testing 0.034±0.037 ng/ml (group 1), modified upper limit of vulnerability testing 0.053±0.025 ng/ml (group 2), safety margin testing 0.094±0.051 ng/ml (group 3). Sample size was planned to obtain a power of 80% for rejection of the null hypotheses of no mean difference in hsTnT increase in pairwise group comparisons assuming means and standard deviations for the groups as derived from the historical data. Pairwise group comparisons using Welch’s t tests with an adjusted significance level of α = 0.05/3 = 0.017 (Bonferroni correction) were planned assuming normally distributed hsTnT changes. Sample size calculation resulted in a total of 118 patients to be required for the comparison of group 2 with group 1, a total of 28 patients for the evaluation of group 3 versus group 1, and a total of 46 patients for the comparison of group 3 and group 2. Taking into consideration an attrition rate of 15% a total of 175 trial participants were planned to be included in a 2:2:1 randomization scheme.

Data analysis was performed using the software packages SAS version 9.3 (SAS Institute Inc., Cary, NC, USA) and R version 3.0.2 (R Foundation for Statistical Computing, Vienna, Austria). The primary endpoint delta hsTnT was calculated as hsTnT after implantation minus hsTnT before implantation. Analysis of the primary endpoint was carried using the full analysis set (FAS), which was defined following the intention-to-treat principle. All patients with valid hsTnT levels were included in the analysis and each patient was analysed in the group he was randomized to irrespective of protocol deviations. Welch’s t-test with Satterthwaite’s approximation for the degrees of freedom was used for pairwise group comparisons on an adjusted level of significance of 0.017 to account for multiple group comparisons (Bonferroni-adjustment). Accordingly, 98.3% confidence intervals for the differences of group means are presented. The primary endpoint was additionally analysed in the per protocol population in the sense of a sensitivity analysis.

Secondary endpoints were analysed in an explorative manner. For categorical outcomes absolute and relative frequencies are presented and group comparisons were performed using chi-squared tests. For quantitative measures means and standard deviations or medians and ranges (minimum to maximum) are shown. Analysis of variance (ANOVA) was used to test for equality of all group means for symmetrically distributed quantitative measures, Kruskal-Wallis tests were performed for skewed data. Pairwise group comparisons were conducted using t-tests or Mann-Whitney U tests, as appropriate. Spearman’s rank correlation coefficient was considered to evaluate associations between quantitative baseline data and change in hsTnT.

## Results

### Patient characteristics

**Fig 1:**
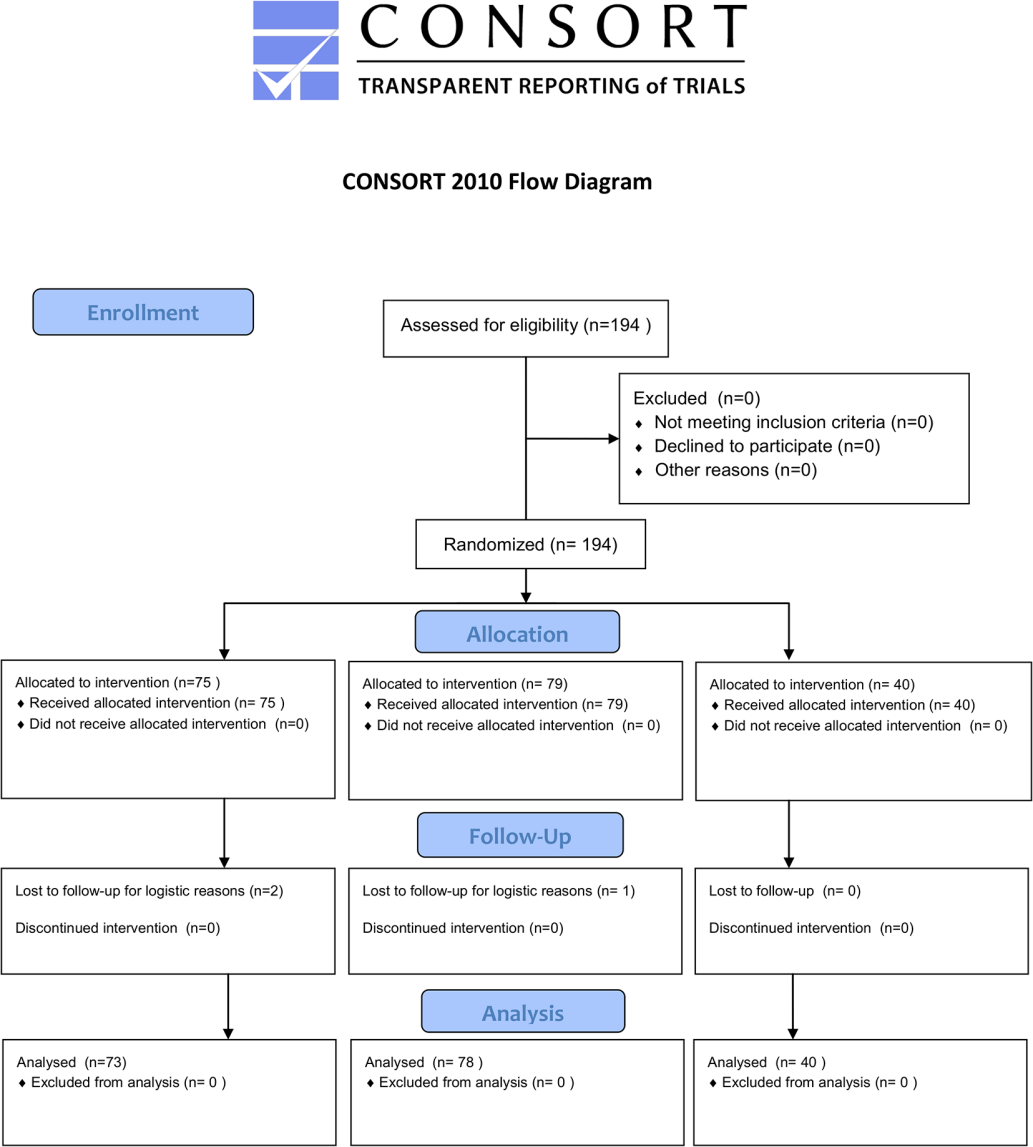
Consort Flow Diagram according to the CONSORT reporting guidelines.

A total of 194 patients were included in three German centres between June 2010 and August 2012. Of these, 75 patients were randomized to *“implantation of an ICD without intraoperative ICD testing”*, 79 patients to “*implantation of the ICD with modified upper limit of vulnerability testing”* and 40 patients to “*ICD implantation with induction of ventricular fibrillation and termination by ICD shock*” (Fig 1). Baseline characteristics for the patients and the procedural data are given in Tables 2 and 3 respectively. They did not differ significantly between the three groups.

**Table 2:** Patient characteristics.

	Total cohort(n = 194)	Implantation only(n = 75)	Upper limit of vulnerability testing (n = 79)	Safety margin testing(n = 40)	p-values
**Age [years], mean ± SD**	64.1±12.5	64.9 ± 13.0	63.7 ± 12.7	63.5 ± 11.4	0.777
**Male gender n (%)**	161 (83)	63 (84)	63 (80)	35 (88)	0.544
**Ischemic cardiomyopathy n (%)**	112 (58)	45 (60)	48 (61)	19 (48)	0.338
**Dilated cardiomyopathy n (%)**	78 (40)	28 (37)	29 (37)	21 (53)	0.217
**Primary prevention n (%)**	184 (95)	70 (93)	76 (96)	38 (95)	0.722
**CRT n (%)**	68 (35)	26 (35)	26 (33)	16 (40)	0.743
**LV-EF [%], mean ± SD**	28.7 ± 8.6	29.6 ± 9.4	28.9 ± 8.5	26.5 ± 6.7	0.185
**Renal insufficiency n (%)**	60 (31)	26 (35)	22 (28)	12 (30)	0.651
**Creatinine [mg/dl]median (min-max)**	1.02 (0.44–4.52)	1.09 (0.50–2.91)	1.01 (0.60–4.52)	0.99 (0.44–2.03)	0.688
**Baseline hsTnT [ng/ml], median (min-max)**	0.018 (0.003–0.712)	0.015 (0.003–0.351)	0.016 (0.003–0.712)	0.023 (0.003–0.136)	0.172

**Table 3:** Procedural data.

	Total number of patients (n = 194)	Implantation only (n = 75)	Upper limit of vulnerability testing (n = 79)	Safety margin testing(n = 40)	p-values
**Subcutaneous position of ICD n (%)**	143 (74)	58 (77)	53 (67)	32 (80)	0.211
**Energy (J) Median (Min–Max)**	52 (0–262)	0 (0–200)	62 (0–200)	62 (25–262)	<0.001
**Cut-to-suture time [min] Median (Min–Max)**	64 (17–338)	60 (20–222)	64 (17–338)	73 (25–256)	0.313
**Fluoroscopy time [min] Median (Min–Max)**	2.5 (0–40)	2.6 (0.2–30)	2.3 (0–37)	3.7 (0.1–40)	0.578
**Contrast dye [ml] Median (Min–Max)**	0 (0–350)	0 (0–350)	0 (0–120)	0 (0–250)	0.899
**Intraoperative right ventricular electrode positioning [n] median (Min-Max)**	1 (1–11)	1 (1–11)	1 (1–9)	1 (1–8)	0.387

### Primary endpoint

**Fig 2:**
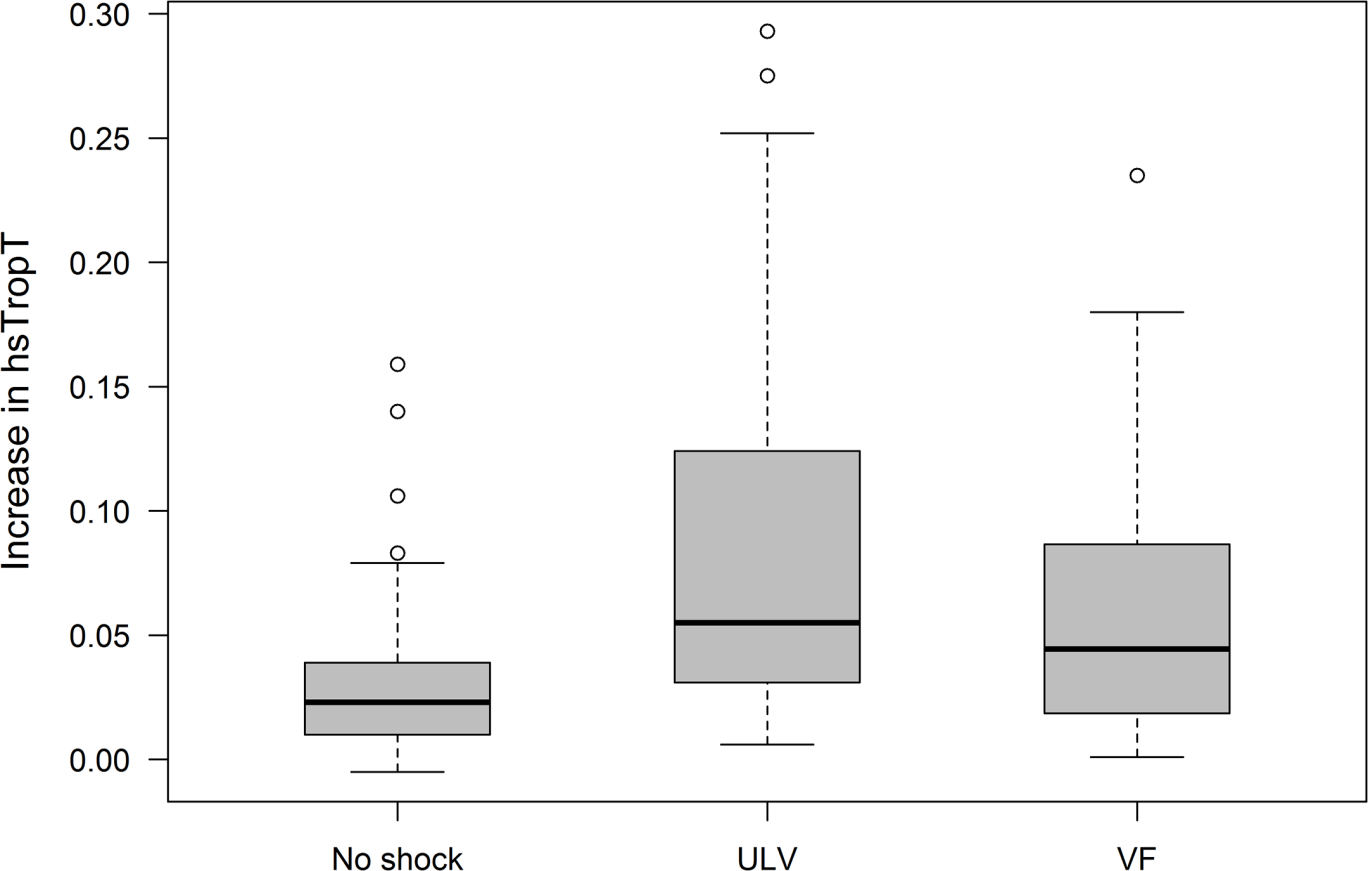
Primary endpoint; increase in hsTnT[ng/ml] (intention-to-treat) for all randomization groups. hsTnT = high sensitive Troponin T, No Shock = Implantation without ICD testing, ULV = Upper Limit of Vulnerability Testing, VF = Induction of Ventricular Fibrillation (traditional safety margin testing).

The primary analysis was performed in the full analysis set including 191 patients; three patients were excluded due to missing hsTnT values for logistic reasons. The myocardial micro-damage assessed by the delta hsTnT was 0.031±0.032 ng/ml in patients without intraoperative ICD-Testing, 0.080±0.067ng/ml in patients with ULV testing and 0.064±0.056ng/ml in patients with safety margin testing. The change in hsTnT was significantly lower in the group without intraoperative testing compared to the group with ULV testing (p<0.001, 98.3% confidence interval for the difference in means [CI] 0.029 to 0.070) and also compared to the group with safety margin testing (p = 0.001, 98.3% CI 0.010 to 0.057). However, delta hsTnT did not differ significantly between the two groups with intraoperative ICD testing (p = 0.179, 98.3% CI -0.013 to 0.044) (Fig 2). Considering a slight deviation of the primary outcome measure from normal distribution, calculations have also been performed with a non-parametric test showing no relevant impact on the significance level.

### Secondary endpoints

**Fig 3:**
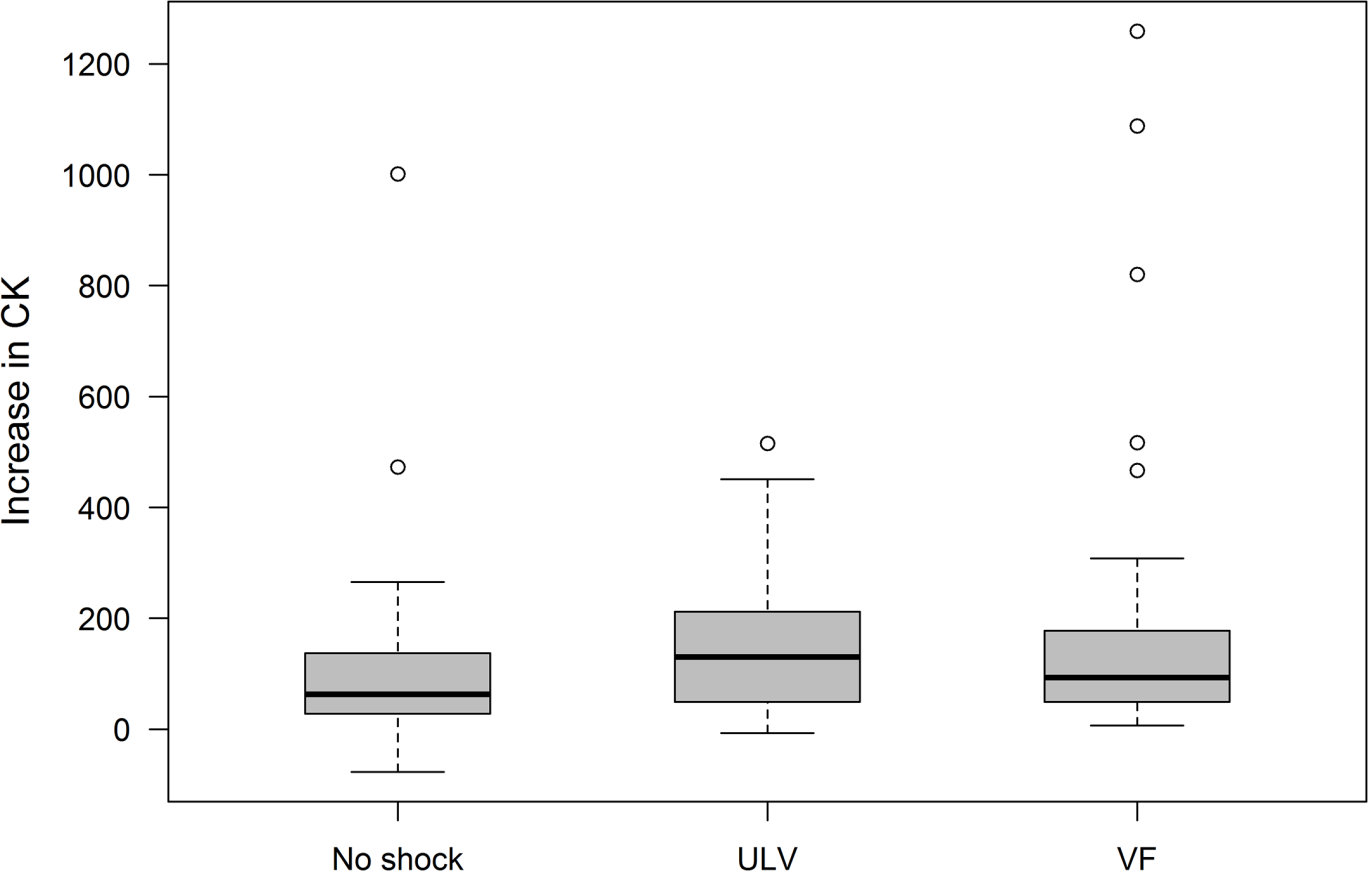
Secondary endpoint; increase in CK [U/l] (intention-to-treat) for all randomization groups. CK = Creatinkinase, No Shock = Implantation without ICD testing, ULV = Upper Limit of Vulnerability Testing, VF = Induction of Ventricular Fibrillation (traditional safety margin testing).

In those patients from the full analysis set with valid CK measures (n = 190) the distribution of delta CK-levels did not differ significantly between patients with intraoperative safety margin testing (median 93U/l (minimum 7 U/l; maximum 1259 U/l)) and the group without such testing (63U/l (-77 U/l; 1001 U/l); p = 0.067) nor was there a significant difference between the group with ULV-testing (130U/l (-7 U/l; 516U/l)) and the group with safety margin testing (p = 0.435). However, there was a significant difference in elevation of postoperative CK-levels between the group of patients with intraoperative ULV-testing and the group without such testing (p = 0.003) (Fig 3).

**Fig 4:**
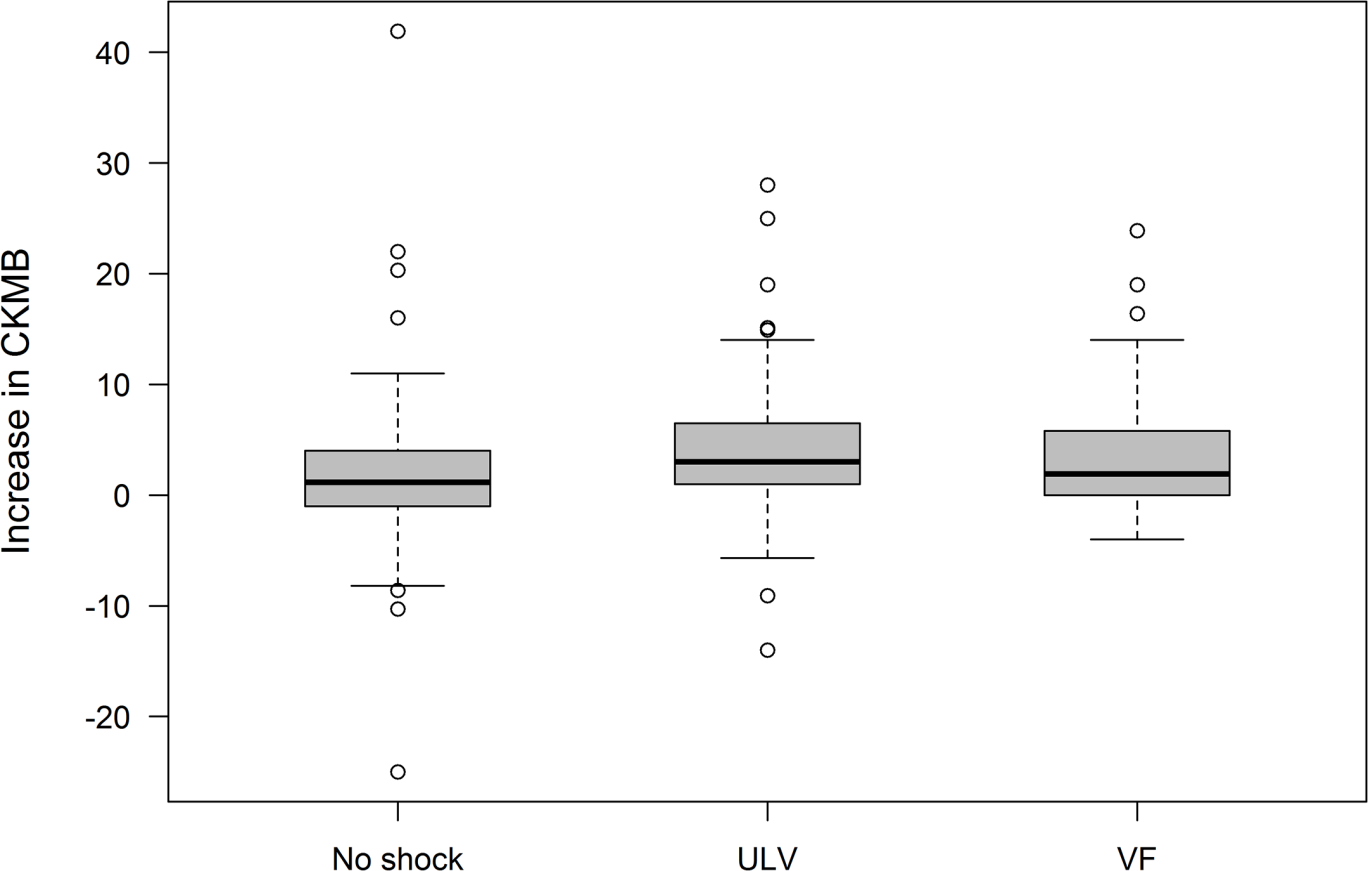
Secondary endpoint; increase in CK-MB[U/l] (intention-to-treat) for all randomization groups. CK-MB = Creatinkinase MB, No Shock = Implantation without ICD testing, ULV = Upper Limit of Vulnerability Testing, VF = Induction of Ventricular Fibrillation (traditional safety margin testing).

Comparing the median delta CK-MB levels between the three groups of patients (n = 178) there was no significant difference in the postoperative elevation of CK-MB levels compared to the initial parameters, when the adjusted level of significance of 0.017 was used (1.2U/l (no testing) vs. 3.0U/l (ULV) p = 0.025; 1.2U/l (no testing) vs. 1.9U/l (safety margin testing) p = 0.313; 3.0U/l (ULV) vs. 1.9U/l (safety margin testing), p = 0.439 (Fig 4).

### Predictors of hsTnT release

Delta hsTnT was not associated significantly with the presence of an ischemic heart disease (p = 0.991) nor did it correlate with the left ventricular ejection fraction (r = 0,110; p = 0.132). There was a weak to moderate correlation between the elevation of hsTnT and the duration of implantation (r = 0.357, p<0.001), the overall fluoroscopy time (r = 0.336, p<0.001) and the number of lead fixations (r = 0.218, p = 0.002). Moreover, for the shock energy delivered on the myocardium, a moderate correlation was observed (r = 0.333, p<0.001). Concerning preoperative creatinine and hsTnT levels, there was no correlation to changes in hsTnT demonstrable (r = -0.108, p = 0.137 and r = -0.096, p = 0.188 respectively).

### Per-protocol analysis

In 37 patients the study protocol could not be performed as intended; (no testing (group 1): n = 10 (13%); ULV (group 2): n = 19 (24%); traditional safety margin testing (group 3): n = 8 (20%)). The main reasons for non-adherence to the protocol were as follows: in group 1 it was the need for safety margin testing due to atypical lead locations (n = 4); in group 2 the reasons were the need for safety margin testing (n = 1) or the unintended induction of VF (n = 13); in group 3 the reasons were an increased DFT (n = 2) or the induction of an arrhythmia other than ventricular fibrillation needing immediate cardioversion (n = 3) or hemodynamic deterioration obviating repeated VF inductions (n = 2). The remaining 157 patients formed the cohort of patients treated exactly according to the protocol.

For this cohort the per-protocol-analysis showed similar results as earlier described above for the intention-to-treat population. There was a significant difference in delta hsTnT six hours after implantation between the group of patients with intraoperative ICD testing and in the group of patients without any testing (0.031±0.033ng/ml (no testing) vs. 0.084±0.071ng/ml (ULV) p<0.001; 0.031±0.033ng/ml (no testing) vs. 0.064±0.060ng/ml (safety margin testing) p = 0.007). Between the two patient groups with intraoperative ICD testing there was no significant difference in delta hsTnT levels (0.084±0.071ng/ml (ULV) vs. 0.064±0.060ng/ml (safety margin testing), p = 0.155).

## Discussion

Serum hsTnT levels in relation to the implantation of an ICD are poorly characterized. The main findings of the randomized Trop-Shock-trial are: (1) The implantation of an ICD is associated with an elevation of serum levels of hsTnT; (2) the study gives reference values of post-operative hsTnT serum levels in relation to different strategies of DFT assessments (non-testing strategy, testing with the mere application of shocks, traditional safety margin testing); (3) there is no significant difference in the post-operative rise of hsTnT levels between a testing strategy that applies shocks but aims to avoid the induction of ventricular fibrillation and the traditional safety margin testing with the repeated induction of ventricular fibrillation followed by defibrillation; (4) the post-operative release of hsTnT is significantly higher in patients with intraoperative ICD testing than in patients without testing at all.

Our results confirm the observed post-procedural elevation of Troponin levels seen in previous studies [7–9, 18]. However, the available data only refer to Troponin T and Troponin I but not to hsTnT which today is commonly used to assess myocardial micro-damage. In addition to this, available data do not reflect different implantation or testing strategies [7–9]. By randomizing patients to different intra-operative ICD testing strategies, individual reference values for each of the testing approaches could be obtained. Post-operative hsTnT levels exceed the reference value for serum hs TnT in a considerable number of patients (Table 2, Fig 2).

Concerning the observed elevation of Troponin levels after ICD shocks, several authors posed the question whether they were related to cardiac arrhythmias or the ICD shock itself [7–8, 10]. This issue has recently been addressed indirectly in an observational study which found elevated troponin levels after inappropriate shocks due to lead failure in otherwise healthy and hemodynamically stable patients [19]. By randomly assigning patients to receive shocks without preceding arrhythmias or to receive shocks to terminate induced ventricular fibrillation, we aimed to more precisely determine the source of myocardial micro-damage. Applying the same amount of energy to the myocardium, there was no statistically significant difference in hsTnT rise between the two groups with shock applications. Thus, we deem that the application of a shock but not the underlying ventricular arrhythmia causes the hsTnT elevation. In this context, electroporation known as an electrically induced dysfunction of myocytes due to transient enhancement of myocyte membrane permeability and disturbed myocyte calcium homeostasis might be of relevance [12,20]. Additionally, damage to the cell membrane due to micro infarction, inflammation or cellular apoptosis may play a role in shock-associated Troponin release [10].

The least rise in post-operative hsTnT levels was observed in the non-testing group which was statistically significantly lower when compared to each of the two groups receiving testing strategies with the application of shocks. This result is in contrast to a recent report by Furniss et al. which suggests that elevation of post-operative hsTnT levels are caused by the implantation and are not enhanced by defibrillation threshold testing [21]. However, the aforementioned study is limited by a small number of included patients and by its non-randomized nature. We therefore believe that minimizing implantation-related myocardial micro-damage is best to be achieved by avoiding shock applications.

Keeping the Troponin release during ICD implantation as low as possible seems to be an intuitive strategy. Such a strategy may be applicable to all ICD recipients as there was no significant correlation between Troponin release and left ventricular ejection fraction or underlying cardiac disease (ischemic versus non-ischemic) in our trial. However, it is unclear whether a lowering of post-operative Troponin levels translates into a favourable long-term outcome of the patients. Therefore, our study cannot give a definitive advice on whether or not to perform ICD testing at implantation. On the one hand, the magnitude of Troponin release has been linked to the patient´s short- and long-term outcome in several clinical circumstances [22–25] and augmented Troponin levels after cardioversion or defibrillation of ventricular tachyarrhythmias have been reported to be associated with increased mortality [10]. On the other hand, these correlations may not necessarily be transferable to the specific setting of elevated hsTnT levels measured after ICD implantation. When randomizing patients to an ICD implantation with or without defibrillation threshold testing, the recent large scale SIMPLE study showed no significant differences between first shock efficacy and mortality in the long run [26]. These results confirmed the observations of the non-randomized SAFE-ICD-Trial [27]. The ongoing NORDIC trial with a similar design may give further insight into the prognosis of patients with or without ICD testing at implantation [28].

Supported by technical improvements of the recent years (e. g. availability of high and ultra-high energy devices, painfree measurements of shock impedance, programmable alternating shock polarity, programming strategies to avoid shocks which make patients less dependent on shocks) these studies may enhance the trend towards a non-testing implantation strategy [29]. Our finding of a significantly larger increase in hsTnT release in the testing groups compared to the non-testing group may provide proponents for a simplified implantation procedure with additional arguments in favour of the discontinuation of routine ICD testing. Currently, the perceptions whether or not to induce ventricular arrhythmias during ICD implantations varies significantly between implanters resulting in different testing strategies and it is anticipated that these divergent practices may only be harmonised after the publication of a consensus statement by the relevant cardiac societies.

Because our trial was designed as an acute study we are not able to conclude on potential long-term adverse effects related to hsTnT release during the ICD implantation procedure. However, our study shows that the ICD shock, not ventricular fibrillation, causes the elevation of hsTnT after defibrillation threshold testing at implantation. When balancing risks and benefits of intraoperative ICD function testing, the myocardial micro-damage inherent to ICD shocks should be considered.

### Limitations

The study was designed to determine myocardial damage in relation to different implantation strategies and aimed to determine whether lead placement, arrhythmia induction or the application of shocks contribute most to a potential myocardial micro-damage. For this, the study protocol was optimized to apply the same cumulative shock energies in the two groups with shock applications. Therefore it was not possible to assess a potential influence of different cumulative shock energies on myocardial damage.

In order to avoid a potential bias in myocardial damage caused by different lead types, only standard active fixation leads were used in the study. It is assumed that this has not influenced the study results in respect to arrhythmia induction or shock application. However, we cannot conclude on the potential differences in myocardial micro-damage related to active versus passive fixation leads and given reference values only refer to active fixation leads.

As the study aimed to characterize the acute effect of different implantation strategies on myocardial damage, no conclusion can be made on a potential long-term impact.

## Conclusion

Myocardial micro-damage assessed by hsTnT is significantly higher in patients with intra-operative defibrillation threshold testing compared to those without testing. Shock applications, with or without arrhythmia induction, did not result in a significantly different increase in hsTnT. Therefore, the ICD shock itself and not ventricular fibrillation seems to cause myocardial micro-damage.

## Supporting Information

S1 CONSORT Checklist Consort Check List according to the CONSORT reporting guidelines. (DOC)

S1 Protocol Study Protocol (German). (DOC)

S2 Protocol Study Protocol (English). (DOC)
