# Supplementary material for: ICD Shock, Not Ventricular Fibrillation, Causes Elevation of High Sensitive Troponin T after Defibrillation Threshold Testing—The Prospective, Randomized, Multicentre TropShock-Trial
Source: PLoS One. 2015 Jul 24;10(7):e0131570. doi: 10.1371/journal.pone.0131570 (PMC4514854; doi:10.1371/journal.pone.0131570)
Supplement: S1 Protocol — (DOC) [file pone.0131570.s002.doc]

# **Studienprotokoll TropSchock-Studie**

# **Verhalten von hs-Troponin nach ICD-Implantation**

**___________________________________________________________________**

**1. Hintergrund:**

Die Therapie der Wahl zur Prophylaxe des plötzlichen Herztods bei Hochrisikopatienten, ist neben der pharmakologischen Therapie die Implantation eines Cardioverters/Defibrillators (ICD).

Üblicherweise erfolgt intraoperativ eine Funktionsprüfung des implantierten Systems, bei welcher Kammerflimmern induziert und die zur Beendigung der Arrhythmie erforderliche Energiemenge abgeschätzt wird. Traditionell gilt die zweimalige Beendigung von induziertem Kammerflimmern mit 10 J unter der maximal verfügbaren Energie des ICD als ausreichende Sicherheitsmarge. Alternativ kann die Defibrillationsschwelle mittels Abgabe von mehreren Schocks in die T-Wellle abgeschätzt werden, ohne dass eine Arrhythmieinduktion erforderlich ist (sogenannte ULV-Testung). Die reine Implantation der ICD-Systeme ohne Testung der Schockfunktionen erscheint bei ausgewählten Patienten und typischer ICD-Systemkonfiguration gerechtfertigt.

Die Notwendigkeit einer spezifischen intraoperativen Testung wird derzeit vor allem wegen des nicht belegten Nutzens der traditionellen Testformen in Frage gestellt (Blatt et al. 2008, Kolb et al. 2009). Zusätzlich erscheint eine Myokardschädigung durch die Arrhythmieinduktion und Schockabgabe möglich (Blendea et al. 2009). Die Auswirkungen einer ICD-Schockabgabe auf das Myokard wurde bislang jedoch unzureichend untersucht.

Retrospektive und nicht-randomisierte Beobachtungsstudien konnten zeigen, dass nach arrhythmiebedingten ICD-Schockabgaben laborchemisch erhöhte Troponinwerte und ein erhöhtes BNP bestehen (Budeus et al., Hasdemir et al, 2002; Hurst et al., 1999; Schlüter et al., 2001). Troponin, ein kardiales Muskelprotein, diente hierbei, wie in der Myokardinfarktdiagnostik etabliert, als Marker einer myokardialen Zellschädigung.

Unklar ist bislang allerdings, woher die Troponinerhöhung nach ICD-Schockabgabe resultiert. Einerseits könnte sie durch den ICD-Schock selbst hervorgerufen sein, andererseits aber auch Zeichen einer kurzzeitigen Myokardischämie durch die zugrunde liegende Arrhythmie sein. Unter Berücksichtigung einer kürzlich gezeigten Korrelation zwischen Troponinerhöhung nach ICD-Schockabgabe und Mortalität (Blendea et al. 2009; Poole et al. 2008), gewinnt die Klärung des Mechanismus der Troponinerhöhung in Hinblick auf die Wahl zukünftiger ICD-Test-Methoden weitreichende Bedeutung. Sollte eine Troponinerhöhung nach ICD-Testung durch die Arrhythmieinduktion verursacht sein, so wäre dies ein weiteres Argument, zukünftig von der traditionellen Defibrillationsschwellentestung abzusehen. Sollte die Troponinerhöhung durch die Schockabgabe des ICD selbst hervorgerufen werden, müssen in Zukunft jegliche ICD-Testungen mit Schockabgabe in Frage gestellt werden.

Ziel der prospektiven und randomisierten TropSchock-Studie ist es daher, zu untersuchen, wie sich Troponin als kardialer Biomarker für eine myokardiale Zellnekrose nach traditioneller ICD-Testung, nach Abschätzung der Defibrillationsschwelle mittels ULV-Testung und nach reiner Implantation ohne Arrhythmieinduktion und Schockabgabe verhält.

**2. Hypothese**:

Nach einer ICD-Implantation mit traditioneller Bestimmung der Defibrillationsschwelle mittels Arrythmieinduktion und Schockabgabe oder mit Schockabgabe in die T-Welle zur Abschätzung der Defibrillationsschwelle ohne Induktion von Kammerflimmern (Upper Limit of Vulnerability) ist hs-Troponin im peripheren Blut gegenüber einer ICD-Implantation ohne Schockabgabe und ohne Kammerflimmerinduktion signifikant erhöht.

**3. Einschlusskriterien**

Alle Patienten zur Neuimplantation eines ICD oder CRT-ICD in typischer Systemkonfiguration (RV-Sonde im Apex, Aggregat linkspectoral) unter Verwendung von Standard-Schraubelektroden.

**4. Ausschlusskriterien**

- akuter Myokardinfarkt oder PCI oder Reanimation in den vorangegangenen 4 Wochen
- herzchirurgische Operation in den vorangegangenen 4 Wochen
- bekannte stenosierte Koronargefäße mit Indikation zur Intervention
- intrakardiale Thromben oder allgemeine Kontraindikation gegen eine Schockabgabe oder Induktion von Kammerflimmern
- atypische Sondenimplantation, so dass eine Bestimmung der Defibrillationsschwelle mittels Kammerflimmerinduktion indiziert ist
- Rechtsseitige Aggregatimplantation
- Geplante externe Elektrokardioversion
- Sondenrevisionen / -extraktionen
- Upgrade eines bestehenden Schrittmachers/ICD
- ASA >= IV
- Teilnahmeverweigerung des Patienten

**5. Studienablauf**

Die Studie ist eine multizentrische, randomisierte, prospektive Studie. Patienten der Klinik für Herz- und Kreislauferkrankungen, bei denen die Implantation eines ICD oder CRT-ICD geplant ist, werden nach Einwilligung zur Teilnahme an der Studie randomisiert für die reine Implantation des Gerätes ohne Schock-Testung, die Implantation des Gerätes mit Schock-Testung nach unten genanntem ULV-Schema oder die Implantation des Gerätes mit traditioneller Testung der Defibrillationsschwelle durch Kammerflimmmerinduktion und Schockabgabe.

1. Randomisierung stratifiziert nach CRT-ICD und Ein-/Zweikammer-ICD für
   1. ICD/CRT-ICD-Implantation mit Kammerflimmerinduktion und Schockabgabe (traditionelles Verfahren zur Bestimmung der Defibrillationsschwelle)

oder

- 1. ICD/CRT-ICD-Implantation mit Schockabgabe nach ULV-Schema (Schema zur Abschätzung der DFT ohne Notwendigkeit einer Arrhythmieinduktion)

oder

- 1. ICD/CRT-ICD-Implantation ohne DFT-Abschätzung

Die Randomisation erfolgt jeweils getrennt nach (Ein-/Zweikammer-)ICD oder CRT-ICD in 5er Blöcken nach einer computergenerierten Randomisationsliste.

1. Blutentnahme

Es erfolgt präoperativ die Bestimmung folgender Laborparameter aus peripherem venösen Blut: Elektrolyte (insb. Kalium), Nierenstatus (Kreatinin, Harnstoff, Clearance), hsTroponin, CK, CK-MB, CK-MB/CK, BNP

1. präoperative Dokumentation folgender Punkte: Begleiterkrankungen (insbesondere kardiale Anamnese (KHK/PCI/Infarkt), linksventrikuläre Pumpfunktion, Nierenerkrankungen, aktuelle Medikation
2. Tiefe Sedierung nach standardisiertem Vorgehen (siehe anhängendes Anästhesie-Protokoll)
3. Implantation des ICD oder CRT-ICD mit Standard-Schraubelektroden
4. ICD-Testung gemäß Randomisierung (Energiewahl für die einzelnen ICD-Schockabgaben siehe „Anhang Schockenergien“)
5. Erfassung prozeduraler Charakteristika (Anzahl der Schraubvorgänge bei den Sondenplatzierungen, OP-Dauer, Durchleuchtungszeit, KM-Menge, retropectorale Aggregatlage, Notwendigkeit einer Kardioversion zur Terminierung von Arrhythmien (🡪 Ausschluss aus Studie und Nachverfolgung in einem Register))
6. Intraoperative Blutentnahme („BNP“) 5 Min. nach Schockabgabe in der „ Schock-Gruppe“ bzw. vor Hautnaht in der „Implantations-Gruppe“
7. Postoperative Blutabnahme 6 Stunden nach intraoperativem ICD-Schock bzw. nach entsprechendem Zeitpunkt in der Kontrollgruppe: Elektrolyte (insb. Kalium), Nierenstatus (Kreatinin, Harnstoff, Clearance) hsTroponin, CK, CK-MB, CK-MB/CK, BNP

**Anhang Schockenergien**

In der Patientengruppe mit traditioneller DFT-Abschätzung (VF+Schock):

Zweimalige Kammerflimmerinduktion mittels Schock-auf-T (1J) und Terminierung mit 10 J Sicherheitsmarge

In der Patientengruppe mit einer DFT-Abschätzung mittels ULV: Schockabgabe nach folgendem Upper-Limit-of-Vulnerability-Schema

Schema: [ ( Max. Energie-10 J) x 2+2]

Die über die Sonden abgegebenen kumulativen Energiemengen entsprechen dabei jenen der traditionellen Testung und werden auf 3 Schockabgaben in die T-Welle verteilt.

| Hersteller  MDT  SJM  BIO  GDT  ELA  Max.Schockenergie  35 J  36 J  40 J  41 J  42 J  **Σ Energie traditionell**  **52 J**  **52 J**  **62 J**  **64 J**  **66 J**  ULV 1  22 J  22,5 J  26 J  27 J  28 J  ULV 2  18 J  17,5 J  22 J  23 J  22 J  ULV 3  12 J  12,5 J  14 J  14 J  16 J  **Σ Energie ULV**  **52 J**  **52,5J**  **62 J**  **64 J**  **66 J** |  |  |  |  |  |
| --- | --- | --- | --- | --- | --- |

**6. Endpunkte**

Primärer Endpunkt: Plasmakonzentration des hs Troponin 6h post Schock

Sekundärer Endpunkt: Plasmakonzentration von CK, CK-MB, CK-MB/Gesamt-CK, BNP

„Prespecified“ sekundäre Endpunkte:

- Korrelation der OP-Dauer mit einer hsTnT-Erhöhung
- Korrelation der EF mit einer hsTnT-Erhöhung
- Korrelation der Sedierungstiefe mit einer hsTnT-Erhöhung
- Korrelation der Anzahl der Schraubvorgänge mit einer hsTnT-Erhöhung
- Korrelation des Koronarstatus mit einer hsTnT-Erhöhung

**7. Studienbedingte Risiken/Komplikationen**

Bei Teilnahme an der Studie wird eine zusätzliche Blutentnahme intraoperativ und 6 Stunden post-Schock/OP notwendig.

**8. Statistik und Fallzahl**

Die Studie ist eine multizentrische, randomisierte, prospektive Studie. Es soll untersucht werden, wie sich hsTroponin nach ICD-Implantation im peripheren Blut verhält. Basierend auf Messungen von hs-Troponin nach ICD-Implantationen wird angenommen, dass ein Anstieg von hsTroponin 6 Stunden post operationem am niedrigsten nach reiner Implantation, mäßiggradig nach ULV-Testung und am höchsten nach traditioneller Bestimmung der Defibrillationsschwelle ausfällt. Folgende δ-Werte von hs-Troponin wurden in einer nicht-randomisierten Pilotphase gemessen:

Gruppe 1 (reine Implantation):    0.0342±0.0366

Gruppe 2 (ULV): 0.053±0.025

Gruppe 3 (traditionelle Testung): 0.094±0.051

Unter der Annahme G2>G1, G3>G1, G3>G2 bei angenommener Normalverteilung mit unterschiedlichen Standardabweichungen, einer Power von 80% und einem Signifikanzniveau bei dreifach zweiseitigem Test von α=0,5/3= 0,01667 werden n=118 für G2>G1, n=28 für G3>G1 und n= 46 für G3>G2 berechnet (student t test). Aufgrund dieser Berechnungen kann eine 2:2:1 Randomisierung durchgeführt werden. Bei einer angenommenen Drop-out Rate von ~ 15% müssen 70 (G1):70(G2):35 (G3) = 175 Patienten in die Studie eingeschlossen werden.

**9. Ort der Studiendurchführung und Principal Investigator**

Sie Studie wird multizentrisch am Deutschen Herzzentrum München, Klinik für Herz- und Kreislauferkrankungen, am Universitätsklinikum Freiburg, Medizinische Klinik III und an der Medizinischen Klinik 1, Landshut-Achdorf durchgeführt. Principal Investigators sind PD Dr. Christof Kolb und Dr. med. Verena Semmler.

**Literatur:**

1. *Budeus M, Salibassoglu E, Schymura AM, Reinsch N, Wieneke H, Sack S, Erbel R.* Effect of Induced Ventricular Fibrillation and Shock Delivery on Brain Natriuretic Peptide Measured Serially Following a Predischarge ICD Test. Indian pacing and Electrophysiology Journal 2007; 7:195-203
2. *Blatt JA, Poole JE, Johnson GW, Callans DJ, Raitt MH, Reddy RK, Marchlinski FE, Yee R, Guarnieri T, Talajic M DJ, Anderson J, Chung K, Wong WS, Mark DB, Lee KL, Bardy GH.* No benefit from defibrillation threshold testing in the SCD-HeFT (Sudden Cardiac Death in Heart Failure Trial). J Am Coll Cardiol 2008; 12;52:551-6
3. *Blendea D, Blendea M, Banker J, McPherson CA.* Troponin T elevation after implanted defibrillator discharge predicts survival. Heart. 2009; 95: 1153-8
4. *Hasdemir C, Shah N, Rao AP, Acosta H, Matsudaira K, Neas BR, Reynolds DW, Po S, Lazzara R, Beckman KJ.* Analysis of Troponin I levels after spontaneous implantable cardioverter defibrillator shocks. J Cardiovasc Electrophysiol. 2002; 13:144-50
5. *Hurst TM, Hinrichs M, Breidenbach C, Katz N, Waldecker B.* Detection of myocardial injury during transvenous implantation of automatic cardioverter-defibrillators. J Am Coll Cardiol. 1999; 34:402-8
6. Kolb C, Tzeis S, Zrenner B. Defibrillation threshold testing: tradition or necessity? Pacing Clin Elektrophysiol. 2009; 32:570-2
7. *Poole JE, Johnson GW, Hellkamp AS.* Prognostic importance of defibrillator shocks in patients with heart failure. N Engl J Med 2008; 359:1009-17
8. *Schlüter T, Baum H, Plewan A, Neumeier D.* Effects of Implantable Cardioverter Defibrillator Implantation and Shock Application on Biochemical Markers of Myocardial Damage, Clin Chem 2001; 47: 459-463
