# Supplementary material for: ICD Shock, Not Ventricular Fibrillation, Causes Elevation of High Sensitive Troponin T after Defibrillation Threshold Testing—The Prospective, Randomized, Multicentre TropShock-Trial
Source: PLoS One. 2015 Jul 24;10(7):e0131570. doi: 10.1371/journal.pone.0131570 (PMC4514854; doi:10.1371/journal.pone.0131570)
Supplement: S2 Protocol — (DOC) [file pone.0131570.s003.doc]

Study protocol TropShock-Trial

1. Background:

In high risk patients the implantation of an implantable cardioverter defibrillator is the therapy of choice for the prevention of sudden cardiac death.

To validate the function of the device defibrillation threshold testing is performed intraoperatively. Therefore, ventricular fibrillation is induced and terminated by the ICD. Traditionally, the termination of the induced arrhythmia with 10 Joule (J) below the maximum output energy of the device is considered to be an adequate safety margin. Alternatively, upper limit of vulnerability testing can be performed. Therefore, several ICD shocks are administered to the ascending part of the T wave without the necessity of inducing ventricular arrhythmias. When implanting the device in a typical configuration the implantation without ICD testing seems also to be justified.

The need for a traditional safety margin testing has been questioned due to its unknown benefit (Blatt et al. 2008, Kolb et al. 2009). Additionally, the induction of ventricular arrhythmias and the application of shocks on the myocardium may cause myocardial damage (Blendea et al. 2009). The effect of an ICD shock on human myocardial tissue has not been studied entirely. Several retrospective and observational studies demonstrated elevated troponin levels and elevated BNP levels after ICD shocks with underlying arrhythmias (Budeus et al., Hasdemir et al, 2002; Hurst et al., 1999; Schlüter et al., 2001). In this context, troponin was used as a marker for myocardial cell damage.

However, the reason for the elevation of troponin after ICD shocks remains unclear. A potential cause of myocardial micro-damage might be that ventricular fibrillation causes myocardial ischemia and consequently an increase in cardiac enzyme levels, or the damage might be directly related to the shock itself. In the context of intraoperative ICD testing the answer to this question gains importance as there have been recent studies demonstrating a correlation of mortality and the elevation of troponin after ICD shocks (Blendea et al. 2009; Poole et al. 2008). If the troponin elevation is caused by the induction of an arrhythmia this would be a further argument to quit traditional defibrillation threshold testing. If the elevation of troponin is related to the ICD shock itself, any ICD testing should be questioned.

Therefore, the prospective, randomized TropShock trial intends to characterize changes in levels of troponin as a cardiac biomarker of myocardial cell necrosis after traditional ICD testing, after ULV testing and after mere implantation.

2. Hypothesis:

The elevation of high-sensitive troponin serum levels after ICD implantation with traditional defibrillation threshold testing or with upper limit of vulnerability testing is significantly higher compared to an implantation procedure without ICD testing.

3. Inclusion criteria:

Patients receiving the de-novo implantation of an (CRT-) ICD for primary or secondary prevention of sudden cardiac death with the left-sided placement of an ICD and the intention to place an active fixation defibrillation lead in the apical region of the right ventricle.

4. Exclusion criteria:

- myocardial infarction, percutaneous coronary intervention, resuscitation or cardiac surgery four weeks prior to ICD implantation,

- coronary artery disease with an indication of revascularization,

- presence of intra-cardiac thrombi,

- contraindication for the induction of ventricular fibrillation or the application of shocks,

- atypical placement of the ventricular lead requiring defibrillation testing,

- right-sided placement of the ICD,

- planned external cardioversion of atrial tachyarrhythmias,

- lead revision or lead extraction,

- upgrade of an pre-existing ICD to a CRT-ICD,

- ASA (American Society of Anesthesiologists) status ≥4,

- inability to give written informed consent, age <18 years.

5. Procedure

The TropShock Trial is a prospective, randomized, multicenter trial. Patients, planned to receive an ICD implantation, are randomly assigned to one of the following three implantation strategies: implantation without arrhythmia induction or shock application, implantation with DFT estimation by a modified upper limit of vulnerability testing including only shock applications or implantation with traditional testing including the induction of ventricular tachyarrhythmias and shocks.

1. Random patient allocation stratified for CRT-ICD or VVI/DDD-ICD

a. ICD/CRT-ICD implantation with induction of ventricular arrhythmias and ICD shocks (traditional procedure for DFT estimation)

or

b. ICD/CRT-ICD implantation with ICD shocks according to the ULV procedure (estimation of the DFT without induction of arrhythmias)

or

c. ICD/CRT-ICD-Implantation without estimation of the DFT

Random patient allocation will be done separately for VVI/DDD-ICD and CRT-ICD in groups of five following a computer-generated randomisation list.

2. Blood sample: The following parameters are determined preoperatively from peripheral venous blood: sodium and potassium, creatinine, urea, clearance, hsTroponin, creatinkinase, CK-MB, CK-MB/CK, BNP

3. Documentation of the following parameters preoperatively: medical history (cardial and other), left ventricular ejection fraction, kidney disease, medication

4. Conscious sedation following a standardised procedure

5. Implantation of the ICD or CRT-ICD using active fixation leads

6. ICD testing according to randomisation (for shock energies please see attachment “shock energies”)

7. Documentation of the following parameters: number of active fixations, time of implantation procedure, fluoroscopy time, amount of contrast dye, device location, necessity of cardioversions to terminate arrhythmias)

8. Intraoperative blood sample (BNP) 5 minutes after ICD shock in the “testing groups” or after suture in the “implantation group”, respectively.

9. Postoperative blood sample six hours after intraoperative ICD shock or after suture, respectively: sodium and potassium, creatinine, urea, clearance, hsTroponin, creatinkinase, CK-MB, CK-MB/CK, BNP

**Shock energies**

For the traditional safety margin testing (VF+shock): Induction of ventricular fibrillation by shock on T (1 J) and termination with 10J below the maximal available energy of the respective ICD. This has to be performed twice.

Estimation of DFT by modified ULV testing according to the following fomula: [ ( Max. Energie-10 J) x 2+2]. The cumulative energy adds up to the same as that which would be delivered cumulatively during traditional 10 J safety margin testing and is distributed to three shocks on T.

| **Manufacturer** |  | **Medtronic** | **St. Jude Medical** | **Biotronik** | **Boston Scientific** | **Sorin** |
| --- | --- | --- | --- | --- | --- | --- |
|  | Maximal shock energy of the device (Joule) | 35 | 36 | 40 | 41 | 42 |
|  | Shock 1 [J] | 1+25 | 1+25 | 1+30 | 1+31 | 1+32 |
| **Safety margin testing** | Shock 2 [J] | 1+25 | 1+25 | 1+30 | 1+31 | 1+32 |
|  | Σ energy [J] | 52 | 52 | 62 | 64 | 66 |
|  | Shock 1 [J] | 22 | 22,5 | 26 | 27 | 28 |
| **Upper limit of vulnerability testing** | Shock 2 [J] | 18 | 17,5 | 22 | 23 | 22 |
|  | Shock 3 [J] | 12 | 12,5 | 14 | 14 | 16 |
|  | Σ energy [J] | 52 | 52,5 | 62 | 64 | 66 |

6. Endpoints

Primary study endpoint: serum high sensitive troponin T levels 6 hours after ICD shock compared to the preoperative baseline value

Secondary endpoint: serum creatinkinase (total and MB fraction) 6 hours after ICD shock compared to the preoperative baseline value

Prespecified endpoints: Correlation of delta high sensitive troponin T levels and the procedure time, the left ventricular ejection fraction, the number of intra-operative lead repositionings, the underlying cardiac disease.

7. Risk and complications:

An additional blood sample will be taken intraoperatively and 6 hours thereafter.

8. Statistics:

The trial is a multicenter, randomized, prospective trial. Based on data derived from the most recent 30 patients it is assumed that an elevation of high sensitive troponin T 6 hours postoperatively is low after mere implantation, moderate after ULV testing and high after traditional ICD testing. The following mean delta ± standard deviations in high sensitive troponin T levels were observed in the non randomized pilot phase:

Group 1 (implantation without ICD testing): 0.0342±0.0366 ng/ml,

Group 2 (modified ULV): 0.053±0.025 ng/ml,

Group 3 (safety margin testing): 0.094±0.051 ng/ml

Sample size was planned to obtain a power of 80% for rejection of the null hypotheses of no mean difference in hsTnT increase in pairwise group comparisons assuming means and standard deviations for the groups as derived from the historical data. Pairwise group comparisons using Welch’s t tests with an adjusted significance level of α = 0.05/3 = 0.01667 were planned assuming normally distributed hsTnT changes. Taking into consideration an attrition rate of 15 % a total of 70(G1):70(G2):35(G3)=175 trial participants were planned to be included in a 2:2:1 randomization scheme.

9. Participating centers and principle investigators

Patients will be included at the Deutsches Herzzentrum München, Klinik für Herz- und Kreislauferkrankungen, Universitätsklinikum Freiburg, Medizinische Klinik III and Medizinische Klinik 1, Landshut-Achdorf. Principle investigators are PD Dr. Christof Kolb and Dr. med. Verena Semmler

Literature:

1. Budeus M, Salibassoglu E, Schymura AM, Reinsch N, Wieneke H, Sack S, Erbel R. Effect of Induced Ventricular Fibrillation and Shock Delivery on Brain Natriuretic Peptide Measured Serially Following a Predischarge ICD Test. Indian pacing and Electrophysiology Journal 2007; 7:195-203

2. Blatt JA, Poole JE, Johnson GW, Callans DJ, Raitt MH, Reddy RK, Marchlinski FE, Yee R, Guarnieri T, Talajic M DJ, Anderson J, Chung K, Wong WS, Mark DB, Lee KL, Bardy GH. No benefit from defibrillation threshold testing in the SCD-HeFT (Sudden Cardiac Death in Heart Failure Trial). J Am Coll Cardiol 2008; 12;52:551-6

3. Blendea D, Blendea M, Banker J, McPherson CA. Troponin T elevation after implanted defibrillator discharge predicts survival. Heart. 2009; 95: 1153-8

4. Hasdemir C, Shah N, Rao AP, Acosta H, Matsudaira K, Neas BR, Reynolds DW, Po S, Lazzara R, Beckman KJ. Analysis of Troponin I levels after spontaneous implantable cardioverter defibrillator shocks. J Cardiovasc Electrophysiol. 2002; 13:144-50

5. Hurst TM, Hinrichs M, Breidenbach C, Katz N, Waldecker B. Detection of myocardial injury during transvenous implantation of automatic cardioverter-defibrillators. J Am Coll Cardiol. 1999; 34:402-8

6. Kolb C, Tzeis S, Zrenner B. Defibrillation threshold testing: tradition or necessity? Pacing Clin Elektrophysiol. 2009; 32:570-2

7. Poole JE, Johnson GW, Hellkamp AS. Prognostic importance of defibrillator shocks in patients with heart failure. N Engl J Med 2008; 359:1009-17

8. Schlüter T, Baum H, Plewan A, Neumeier D. Effects of Implantable Cardioverter Defibrillator Implantation and Shock Application on Biochemical Markers of Markers of Myokardial damage, Clin Chem 2001; 47: 459-463
